# Innovative COVID-19 Point-of-Care Diagnostics Suitable for Tuberculosis Diagnosis: A Scoping Review

**DOI:** 10.3390/jcm13195894

**Published:** 2024-10-02

**Authors:** Lydia M. L. Holtgrewe, Sonal Jain, Ralitza Dekova, Tobias Broger, Chris Isaacs, Grant Theron, Payam Nahid, Adithya Cattamanchi, Claudia M. Denkinger, Seda Yerlikaya

**Affiliations:** 1 Department of Infectious Diseases and Tropical Medicine, Heidelberg University Hospital and Faculty of Medicine, Heidelberg University, 69120 Heidelberg, Germany; sonal.jain@uni-heidelberg.de (S.J.); ralitza.dekova@uni-heidelberg.de (R.D.); tobias.broger@uni-heidelberg.de (T.B.); claudia.denkinger@uni-heidelberg.de (C.M.D.); seda.yerlikaya@uni-heidelberg.de (S.Y.); 2 Connected Diagnostics Limited, London WC2H 9JQ, UK; chris.isaacs@connected-dx.com; 3 DSI-NRF Centre of Excellence for Biomedical Tuberculosis Research, South African Medical Research Council Centre for Tuberculosis Research, Division of Molecular Biology and Human Genetics, Faculty of Medicine and Health Sciences, Stellenbosch University, P.O. Box 241, Cape Town 8000, South Africa; gtheron@sun.ac.za; 4 UCSF Center for Tuberculosis, University of California San Francisco, San Francisco, CA 94143, USA; pnahid@ucsf.edu (P.N.); adithya.cattamanchi@ucsf.edu (A.C.); 5 Division of Pulmonary Diseases and Critical Medicine, University of California Irvine, Irvine, CA 92697, USA; 6 German Center for Infection Research (DZIF), Partner Site Heidelberg University Hospital, 69120 Heidelberg, Germany

**Keywords:** tuberculosis, COVID-19, rapid diagnostic tests, point-of-care testing, missed diagnosis

## Abstract

Rapid and accurate point-of-care (POC) tuberculosis (TB) diagnostics are crucial to bridge the TB diagnostic gap. Leveraging recent advancements in COVID-19 diagnostics, we explored adapting commercially available POC SARS-CoV-2 tests for TB diagnosis in line with the World Health Organization (WHO) target product profiles (TPPs). A scoping review was conducted following PRISMA-ScR guidelines to systematically map POC antigen and molecular SARS-CoV-2 diagnostic tests potentially meeting the TPPs for TB diagnostic tests for peripheral settings. Data were gathered from PubMed/MEDLINE, bioRxiv, medRxiv, publicly accessible in vitro diagnostic test databases, and developer websites up to 23 November 2022. Data on developer attributes, operational characteristics, pricing, clinical performance, and regulatory status were charted using standardized data extraction forms and evaluated with a standardized scorecard. A narrative synthesis of the data is presented. Our search yielded 2003 reports, with 408 meeting eligibility criteria. Among these, we identified 66 commercialized devices: 22 near-POC antigen tests, 1 POC molecular test, 31 near-POC molecular tests, and 12 low-complexity molecular tests potentially adaptable for TB. The highest-scoring SARS-CoV-2 diagnostic tests were the near-POC antigen platform LumiraDx (Roche, Basel, Switzerland), the POC molecular test Lucira Check-It (Pfizer, New York, NY, USA), the near-POC molecular test Visby (Visby, San Jose, CA, USA), and the low-complexity molecular platform Idylla (Biocartis, Lausanne, Switzerland). We highlight a diverse landscape of commercially available diagnostic tests suitable for potential adaptation to peripheral TB testing. This work aims to bolster global TB initiatives by fostering stakeholder collaboration, leveraging SARS-CoV-2 diagnostic technologies for TB, and uncovering new commercial avenues to tackle longstanding challenges in TB diagnosis.

## 1. Introduction

As healthcare systems gradually recover from the COVID-19 pandemic, tuberculosis (TB) remains the world’s leading infectious killer, with 1.6 million new cases and 1.3 million deaths in 2022 alone [1]. Despite a modest 8.7% reduction in TB incidence between 2015 and 2022, achieving the World Health Organization (WHO) End TB Strategy’s 50% reduction target by 2025 is still distant [1]. To reach this milestone and curb community transmission, closing the TB diagnostic gap is essential [2]. Sputum smear microscopy, commonly used in low-resource settings due to its rapid results and cost-effectiveness, suffers from low sensitivity, leading to missed TB cases [3]. WHO-recommended rapid diagnostic tests (WRDs) offer higher accuracy and the capability to detect drug resistance, even in decentralized settings. However, widespread adoption of WRDs is impeded by high costs and maintenance requirements [3]. As a result, only 47% of TB cases reported in 2022 were diagnosed using WRDs [4,5,6]. Bridging the TB diagnostic gap requires improved access to and utilization of rapid, accurate, and affordable point-of-care (POC) diagnostic tests and better linkage to treatment [7].

To guide developers toward fit-for-purpose TB diagnostics, the WHO defined high-priority target product profiles (TPP) in 2014, with a revision published in August 2024 [8,9]. Current WRDs fail to meet TPP requirements for peripheral settings due to their reliance on sputum, inadequate performance, high cost, and operational limitations [8,10,11]. Fit-for-purpose peripheral TB diagnostic tests meeting TPP criteria are needed to achieve the WHO’s goal of 100% global WRD coverage [6].

Increased funding and collaborative efforts, such as the Access to COVID-19 Tools Accelerator and RADx, have driven significant growth in diagnostic R&D, resulting in diverse diagnostic products for remote and at-home testing [12,13]. As the COVID-19 diagnostics market declines, developers are exploring new applications for their technologies [12]. TB is a promising choice due to its substantial disease burden, supportive government initiatives, and in-kind funding for validation through established research networks. Given the similarities in transmission through airborne infectious aerosols and droplets, primary pulmonary involvement, and replication sites, there is potential for applying current COVID-19 diagnostic tests to TB. Shared potential sample types, such as oral swabs further support the feasibility of using similar diagnostic approaches [3,14]. These shared characteristics suggest that diagnostic technologies developed for COVID-19 might be adapted for TB detection, potentially enhancing diagnostic efficiency and accessibility.

This scoping review systematically maps commercially available POC antigen and molecular SARS-CoV-2 diagnostics that could meet the TPP for TB diagnostics [8]. It aims to identify promising innovations to facilitate interactions among device and assay developers and other key stakeholders, leveraging COVID-19-driven diagnostics to address the TB diagnostic gap.

## 2. Methods

This scoping review examined the scientific literature, SARS-CoV-2 test databases, and information from developers, following PRISMA Extension for Scoping Reviews (PRISMA-ScR) guidelines (see Table S1) and Levac et al.’s methodological framework [15,16]. We previously published the protocol for this review [17]. Because of the vast literature, we split the review into two parts: this publication focuses on commercialized diagnostics, while a forthcoming publication will focus on tests in development or pre-commercialization.

### 2.1. Definitions and Eligibility Criteria

Definitions and eligibility criteria are defined in the protocol [17]. Due to infrequent reporting on ‘minimal biosafety requirements’, this parameter was omitted from data collection. We introduced the following sub-categories for peripheral in vitro diagnostic (IVD) tests [18]:

POC tests: Tests performed at or near the site of patient care and designed to be instrument-free, disposable, and independent of specific infrastructure (e.g., mains electricity, laboratory equipment, or a cold chain). They require no special skills to administer. Example: Determine^TM^ TB LAM Ag Test (Abbott, Abbott Park, IL, USA).

Near-POC tests: Tests that may be instrument-free or instrument-based but require minimal infrastructure, such as mains electricity for recharging batteries or operating. They can be used in healthcare settings without laboratories and require basic technical skills, such as simple pipetting and sample transfer that do not require precise timing or volumes, to administer. Ideally, they come with pre-set volume transfer pipettes. Example: GeneXpert Edge (Cepheid, Sunnyvale, CA, USA).

Low-complexity tests: Instrument-based tests intended for use in healthcare settings with basic laboratory infrastructure and mains electricity. They require basic technical skills and laboratory equipment, including pipettes, vortex mixers, heating devices, freezers, and/or separate test tubes. Examples: Truenat (Molbio Diagnostics, Nagve, India), GeneXpert 6-/10-color platforms (Cepheid, Sunnyvale, CA, USA).

### 2.2. Information Sources

We initially searched PubMed/MEDLINE for the peer-reviewed literature and bioRxiv and medRxiv for pre-prints [17]. Additional information on tests identified through these databases was obtained from the IVD databases and developer websites listed in the protocol [17]. The China National Medical Products Administration and Indian Central Drugs Standard Control Organization databases were not searched because of limited search function and language barriers [19,20]. The European Database on Medical Devices was inaccessible for data search at the time of data collection [21].

### 2.3. Search

Table 1 in the protocol shows the PubMed/MEDLINE search term [17]. It was adapted for bioRxiv and medRxiv using the medrxivr package in R (version 4.0.5; R Foundation for Statistical Computing) (see Supplementary Methods, Section S1). No restrictions were imposed on the publication date or language.

### 2.4. Selection of Sources of Evidence

Retrieved articles were collated using Covidence software (Veritas Health Innovation, Melbourne, Australia, available online: https://www.covidence.org/, accessed on 29 September 2024), which automatically removes duplicates [22]. Two reviewers (S.Y., L.H.) independently screened titles and abstracts against eligibility criteria, followed by full-text screening using the same software. Discrepancies were resolved through consensus.

### 2.5. Data Charting

We used two Google forms for data charting, developed by one reviewer (S.Y.) and revised in an iterative process by both reviewers (S.Y., L.H.). One reviewer (L.H.) charted information from peer-reviewed articles and pre-prints (see Table S2). For studies mentioning multiple tests, one record per test was charted. Additional information on tests identified in the included articles was collected using the second form from developer websites and IVD databases (see Table S3) [17]. Results tables were cross-checked by a second reviewer (S.J. and R.D.). Data charted from various sources were collated on separate Excel sheets for each diagnostic test.

### 2.6. Variables

We abstracted data on test description, operational characteristics, pricing, performance, and commercialization status, as listed and defined in Table 2 in the study protocol [17].

### 2.7. Synthesis of Results

A narrative synthesis detailing major aspects of included tests like developer information, test characteristics, and clinical performance stratified by technology type (antigen and molecular) and test categories (low-complexity, near-POC, and POC) is provided in the text and tables.

We modified Lehe et al.’s standardized scorecard for evaluating operational characteristics of POC diagnostic devices to match the requirements of the 2014 TPPs, also taking the draft version of the revised TPPs available at the time of data analysis into account [23]. Each test received an overall score ranging from 0 to 110 points based on 22 scoring criteria across seven categories. Each scoring criterion was assigned 1 to 5 points, with missing information scored as one point. A simplified version of the adapted scorecard is shown in Table 1 (See Table S4 for the detailed scoring methodology). Two reviewers (L.H., S.J.) independently scored each device, with conflicts resolved through consensus and discussion with a third reviewer (S.Y.). We present the highest-scoring tests’ characteristics and performance in tables, figures, and text.

**Table 1:** Simplified version of the scorecard (adapted from Lehe et al.) [23].

Scoring Category	Scoring Criteria	Scoring Variables
Category 1: POC features of equipment	Technical specifications	1 Instrument size
		2 Instrument weight
		3 Power requirements
		4 Instrument-free
		5 Connectivity (data export options)
	Data analysis	6 Integrated data analysis
		7 Integrated electronics and software
	Testing capacity	8 Time-to-result
		9 Hands-on-time
		10 Throughput capacity
Category 2: POC features of test consumables	Operating conditions	11 Operating Temperature
		12 Operating Humidity
	Storage conditions	13 Shelf life
Category 3: Ease of use	End user requirements	14 Potential end-user
		15 Number of Manual Sample Processing Steps
Category 4: Performance *	Analytical and clinical performance (COVID-19)	16 Limit of detection (LoD)
		17 Clinical sensitivity
		18 Clinical specificity
Category 5: Cost	Upfront and user costs	19 Capital cost of equipment
		20 Consumable cost
Category 6: Platform versatility	Multi-use ability	21 Applicability of platform to pathogens other than SARS-CoV-2
Category 7: Parameters	Test parameters	22 Number of test parameters available for scoring

**Abbreviations**: PoC = Point-of-Care. Legend: * We only considered independently reported study estimates for scoring purposes. Developer-reported estimates were not used.

## 3. Results

### 3.1. Selection of Sources of Evidence

**Figure 1:**
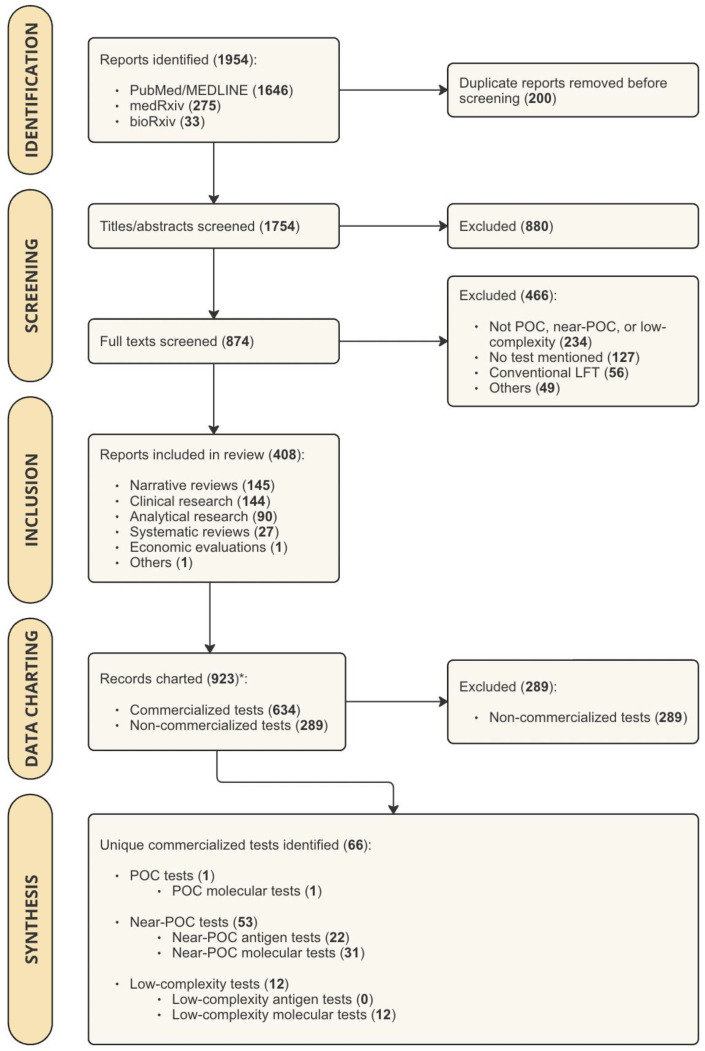
PRISMA Flow Chart showing the results of study search and screening procedures. Abbreviations: POC = point-of-care; LFT = Lateral Flow Test. Legend: * For studies that mentioned more than one test, multiple records were charted (one record per test).

The literature search yielded 1954 results, which were imported into Covidence for screening. After removing 200 duplicates, 1754 articles underwent title/abstract screening. Of these, 874 were eligible for full-text screening. Common reasons for exclusion included assays and instruments not designed for peripheral settings (*n* = 234), no mention of specific tests (*n* = 127), and reporting on conventional LFAs without reading devices or enhanced detection technologies (*n* = 56) (Figure 1). Ultimately, 408 articles were included, identifying 66 commercialized diagnostic tests.

### 3.2. Characteristics of Sources of Evidence

The main sources of evidence were clinical research papers, systematic reviews, and narrative reviews. Clinical performance data were charted from clinical research papers and systematic reviews. Narrative reviews provided details on platform and assay characteristics, occasionally including cost. Developer websites supplemented technical data with information on the test workflow and end-user requirements. The FIND COVID-19 Test Directory provided information on regulatory status, validated assay targets, sample types, and links to country-specific IVD databases for authorization documents such as Instructions for Use. Table S5 offers detailed information on the variables charted from all sources of evidence.

### 3.3. Synthesis of Results

**Figure 2:**
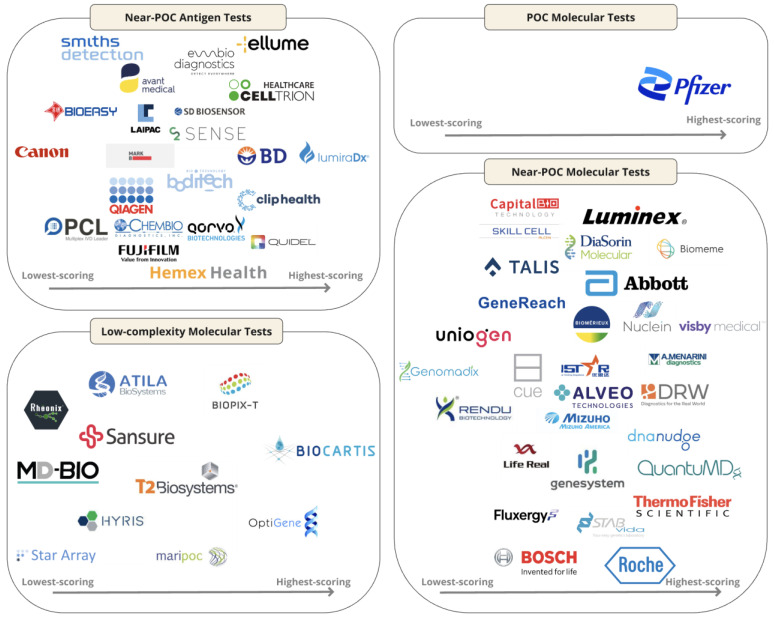
Logo chart illustrating the 63 manufacturers of the 66 included diagnostic tests, sorted by test performance as indicated in the scorecard. Abbreviations: PoC = Point-of-Care.

**Figure 3:**
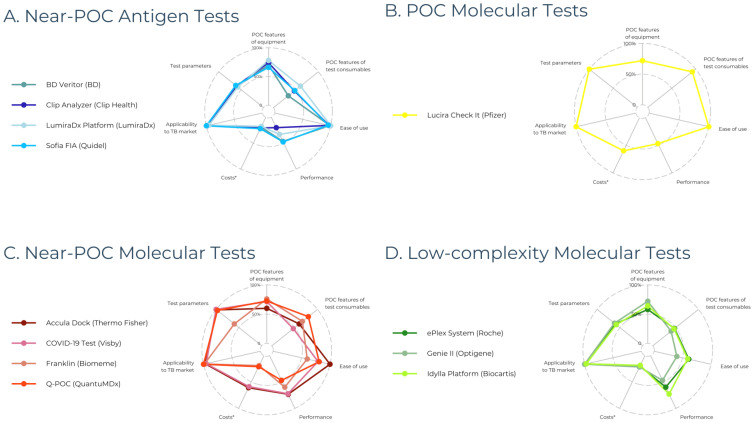
Scorecard performance of the three highest-scoring diagnostic devices across the seven scoring categories, expressed as percentages. (**A**) Four near-POC antigen tests are displayed because BD Veritor, Clip Analyzer, and Sofia FIA received the same score. (**B**) Only one POC molecular test is shown since we only identified one test in this category. (**C**,**D**) The three highest-scoring near-POC and low-complexity molecular tests are shown, respectively. Abbreviations: PoC = Point-of-Care. Legend: * costs (including capital costs and consumable costs) were not reported for most diagnostic tests, resulting in a score of 1/5 (displayed as 20%).

Among the 66 commercialized POC diagnostic tests for SARS-CoV-2, we identified 22 near-POC antigen tests, 1 POC molecular test, 31 near-POC molecular tests, and 12 low-complexity molecular tests. By definition, no POC antigen tests were included, as conventional instrument-free LFTs were excluded. The 63 manufacturers of included diagnostic tests are displayed in Figure 2. Developer and product characteristics, regulatory status, and clinical performance of included diagnostic tests are shown in Tables S6–S9. Table 2 presents the characteristics of the highest-scoring diagnostic test in each test category. Figure 3 displays scores across the seven categories for the three highest-scoring tests in each test category.

**Table 2:** Characteristics of highest-scoring diagnostic devices stratified by test category.

Technology Classification			Antigen Tests	Molecular Tests		
Test Classification			Near-POC	POC	Near-POC	Low-Complexity
Developer, Product Name			LumiraDx, LumiraDx	Pfizer, Lucira Health	Visby, COVID-19 Test	Biocartis, Idylla^TM^
Overall Score *^,†^			73/110 (66%)	83/110 (75%)	78/110 (71%)	66/110 (60%)
Test summary	Platform: Specifications	Dimensions (cm)	21 × 9.7 × 7.3	19.1 × 8.0 × 5.2	13.8 × 6.7 × 4.4	30.5 × 19 × 50.5
		Weight (g)	1100	150	NR	18,600
		Power-supply	Integrated battery (20 tests)	AA batteries	Mains electricity (power adapter)	Standard electricity
		Connectivity	LumiraDx Connect cloud-based services; 2× USB ports; RFID reader; Bluetooth connectivity	None	None	USB port; Direct RJ45 Ethernet cable; Idylla Visualizer (PDF viewer); Idylla Explore (cloud)
		Max. operating temperature (°C)/humidity (%)	30/90	45/95	30/80	30/80
		Multi-use ^‡^	Yes	Yes	Yes	Yes
		Throughput capacity	1	1	1	8
		Costs (USD)	NR	Not applicable (instrument-free)	Not applicable (instrument-free)	NR
	COVID-19 Assay: Specifications	Sample type	NS, NPS	NS	NS, NPS	NPS
		Hands-on time (min)	1	1	<2	<2
		Running time (min)	12	30	30	90
		Shelf-life (months)	NR	18	NR	NR
		Costs per test (USD)	NR	>10/test	NR	NR
		LOD (copies/mL)	NR	900	100–1112	500
		Sensitivity (%)/Specificity (%)	82.7/96.9	93.1/100.0	100.0/98.7	100.0/100.0
POC features of equipment	Score		**39/50 (78%)**	**36/50 (72%)**	**36/50 (72%)**	**32/50 (64%)**
	Pros		Compact instrument size and weight Short test preparation and running time Battery-powered Fully integrated platform with cloud and EHR connectivity Fully integrated data analysis	Compact instrument size and weight Short test preparation and running time Battery-powered Instrument-free, fully integrated platform Fully integrated data analysis	Compact instrument size and weight Short test preparation and running time Instrument-free, fully integrated platform Fully integrated data analysis	High throughput capacity (*n* = 8) Fully integrated platform with multiple options for data export and connectivity Fully integrated data analysis Short test preparation time
	Cons		Low throughout capacity for an instrument-based assay (*n* = 1)	No connectivity	No connectivity Runs on mains electricity only	Large instrument size and heavy weight Runs on mains electricity only
POC features of consumables	Score		**9/15 (60%)**	**14/15 (93%)**	**7/15 (47%)**	**7/15 (47%)**
	Pros		High operating humidity	High operating temperature and humidity Long shelf-life	None	None
	Cons		Unknown shelf-life	None	Unknown shelf-life Low operating temperature	Unknown shelf-life Low operating temperature
Ease of use	Score		**10/10 (100%)**	**10/10 (100%)**	**8/10 (80%)**	**6/10 (60%)**
	Pros		Can be used by community or lay worker without technical skills No manual sample processing steps	Can be used by community or lay worker without technical skills, suitable for self-testing No manual sample processing steps	Can be used by community or lay worker without technical skills, suitable for self-testing Minimal sample processing steps	Can be used by healthcare workers with a minimum of training
	Cons		None	None	None	Sample processing steps require laboratory equipment (pipettes, vortex)
Performance	Score		**5/15 (33%)**	**7/15 (47%)**	**11/15 (73%)**	**11/15 (73%)**
	Pros		None	High study-reported specificity	High study-reported sensitivity and specificity	High clinical sensitivity and specificity
	Cons		No LoD reported Poor study-reported sensitivity Discrepancy between developer-reported and study-reported sensitivity data	High LoD Low study-reported sensitivity	High LoD	High LoD
Cost	Score		**2/10 (20%)**	**6/10 (60%)**	**6/10 (60%)**	**2/10 (20%)**
	Pros		None	No instrument costs	No instrument costs	None
	Cons		Costs not transparently reported	High consumable costs	Consumable costs not transparently reported	Costs not transparently reported
Platform versatility	Score		**5/5 (100%)**	**5/5 (100%)**	**5/5 (100%)**	**5/5 (100%)**
	Pros		Multi-use platform	Multi-use platform	Multi-use platform	Multi-use platform
	Cons		None	None	None	None

Abbreviations: POC = point-of-care; NR = Not Reported; NS = Nasal Swab; NPS = Nasopharyngeal Sample; LoD = Limit-of-Detection. Legend: * color coding: red = overall score/category score <33.3%; orange = overall score/category score <66.6%; green = overall score/category score ≥ 66.6%; ^†^ overall score: calculated by summing the seven category scores; category scores: calculated by aggregating the scores for each variable within the respective scoring category. Please refer to Table S4 for the detailed scoring methodology; ^‡^ multi-use is defined as the ability to analyze multiple biomarkers from one sample on a single diagnostic device (e.g., respiratory panels).

### 3.4. Near-POC Antigen Tests

We identified 22 near-POC antigen tests, including 9 reader-based LFTs, 11 automated immunoassays, and 2 biosensors. The LFT COVID-19 Home Test (Ellume Health, East Brisbane, Australia) was included in this category because result interpretation requires a mobile phone. The highest-scoring test, LumiraDx (LumiraDx, Stirling, UK, recently acquired by Roche Diagnostics, Basel, Switzerland) [24], is a multi-use, microfluidic immunofluorescence assay for detecting antigens in nasal swab (NS) and nasopharyngeal samples (NPS).

### 3.5. POC Molecular Tests

We identified one POC molecular test: Lucira Check It COVID-19 Test (Pfizer, New York, NY, USA), which operates on two AA batteries and is disposable, used for detecting RNA in NS samples.

### 3.6. Near-POC Molecular Tests

This review includes 31 near-POC molecular tests: 25 based on PCR and 6 on isothermal amplification. Most were tabletop platforms, with some handheld platforms like Cue Reader (Cue, Walnut Creek, CA, USA), DoctorVida Pocket Test (STAB Vida, Coimbra, Portugal), and Accula Dock (Thermo Fisher Scientific, Waltham, MA, USA). One disposable molecular test, Visby COVID-19 Test (Visby, San Jose, CA, USA), was included in this category due to its requirement for mains electricity.

### 3.7. Low-Complexity Molecular Tests

The twelve identified low-complexity molecular tests were based on PCR (eight tests) or isothermal amplification technology (four tests). The highest-scoring test in this category was Idylla^TM^ (Biocartis, Lausanne, Switzerland), a multi-use tabletop platform for detecting RNA in NPS, weighing 18 kg and running on mains electricity.

## 4. Discussion

In this comprehensive scoping review, we identified 66 commercially available antigen and molecular tests for diagnosing SARS-CoV-2 at POC and assessed their applicability to TB. Our findings reveal a diverse array of diagnostic tests that hold potential for peripheral TB diagnostic testing.

### 4.1. Antigen Tests

The identified antigen detection platforms excel in compact design, portability, and rapid turnaround times, making them suitable for peripheral settings. The front-runner, LumiraDx, is notable for its quick turnaround, battery operation, and data export options, though it operates within limited temperature ranges [25]. Developers must consider high temperatures and humidity in TB-endemic countries. Harsh environmental conditions may increase technical failures and result in enhanced utilization of maintenance services, resulting in delayed or missed diagnosis in remote settings where technical staff may not be readily available. Additionally, many tests support multi-disease testing, challenging siloed programs and facilitating differential diagnosis [3].

The tests included in this review may meet TPP sensitivity targets for TB detection by employing signal-amplifying technologies, such as LFAs with readers and automated immunoassays utilizing sensitive detection methods like fluorescence and electrochemical approaches. LumiraDx shows promising performance with a LoD of 2–56 PFU/mL for SARS-CoV-2 and a clinical sensitivity of 82.2% [26]. Overall, clinical sensitivity of SARS-CoV-2 assays varies widely, with limited LoD data, complicating the assessment of their potential to detect low-abundance TB antigens like lipoarabinomannan (LAM) in urine that likely requires a LoD in the low pg/mL range to detect TB in all patient groups [27]. Moreover, none of the identified platforms reported the use of urine samples. As a result, successful application to TB will require optimized sample pre-treatment, specific anti-LAM antibodies, and sensitive readout approaches [28,29].

### 4.2. Molecular Tests

The identified molecular tests feature novel assay technologies and platform attributes designed to enhance user-friendliness and testing capacity. These features include easy handling, self-testing options, rapid turnaround times, and the ability to detect multiple pathogens. For instance, at-home molecular tests such as the Visby COVID-19 Test, Lucira Check It, and Cue Reader show considerable promise for use in decentralized due to their compact size, quick results, and ease of use.

Some operational limitations may, however, hinder their widespread adoption in peripheral settings. For example, the Visby COVID-19 Test and Accula Dock require mains electricity, which may affect implementation in areas with unstable power supplies [30]. Although Lucira Check It and Cue Reader use AA batteries or smartphone power, any reliance on smartphones may still pose limitations. Further, most tests, including Lucira Check It and Visby COVID-19 Test, lack adequate data export options, increasing reliance on Wi-Fi and risking human error and data loss. Therefore, adapting these tests for areas with limited infrastructure is essential [31]. As highlighted earlier, their performance in high temperature and humidity conditions typical of TB-endemic regions should also be considered.

Most near-POC platforms have low daily sample throughput and limited multi-use capacity, which can hinder parallel sample analysis and potentially delay treatment delay. Among these, Franklin^TM^ (Biomeme, Philadelphia, PA, USA) stands out with its ability to detect up to 27 targets in 9 samples per PCR run. Multi-disease panels are crucial for integrated public health interventions and should be prioritized in developing novel diagnostics [32]. The WHO’s essential diagnostic list strongly advocates for co-testing of common comorbidities such as TB, HIV, and respiratory pathogens [33]. By incorporating priority diseases into multi-pathogen platforms, testing processes can be streamlined, thus reducing the costs associated with expanding disease coverage [34,35]. These platforms have the potential to significantly enhance disease surveillance and management, especially in resource-limited settings. By offering comprehensive diagnostic coverage, they can enable early detection and treatment of co-infections, ultimately improving overall health outcomes [34]. Alternatively, multiplex capacities could be leveraged to enhance drug susceptibility testing.

The low-complexity GENIE^®^ II (Optigene, Horsham, UK) holds the potential to bridge gaps left by current WRDs [36]. It is battery-powered, operates at high temperatures, delivers results in 30 min for 16 samples, and supports USB data export. However, the need for extra equipment for sample pre-treatment limits its peripheral deployment, though simplifying this process could improve its usability. Conversely, other highly ranked low-complexity platforms, ePlex System (Roche Diagnostics, Switzerland) and Idylla^TM^, do not require sample pre-treatment but need continuous power and only operate at temperatures up to 30 °C, with longer turnaround times (90 to 120 min) for 3 to 8 samples. Their multiplexing capacity and high throughput are better suited for urban centers with substantial test volumes and laboratory infrastructure, similar to GeneXpert Dx [28].

Adapting these molecular platforms for TB poses technical challenges due to *Mycobacterium tuberculosis* (*Mtb*)’s complex cell wall and low bacterial loads in clinical samples [10]. Moreover, the need for sputum processing may not be compatible with these platforms and could affect MTB DNA detection accuracy. However, recent research offers a promising alternative: tongue swabs, when paired with mechanical lysis and sensitive detection methods, can achieve nearly the same sensitivity as sputum in symptomatic patients with low to high sputum bacillary loads [37]. Most identified near-POC molecular tests support oral swabs, suggesting easier adaptability for TB. Other alternative sample types, such as breath aerosols (XBA) and stool samples in children, show potential but still fall short in sensitivity compared to sputum testing [38,39]. While these sample types may be less sensitive than sputum, their ease of collection could improve diagnostic yield, enhance test accessibility and patient acceptability, and reduce overprescribing of empiric antibiotics [40,41,42,43].

However, additional manual processing steps—such as dissolving filters from face masks or heating samples to inactive pathogens—followed by mechanical lysis techniques, like bead beating or sonication to break down *Mtb*, even when non-sputum samples are used for TB detection [37,43]. These steps could create challenges for integration into the molecular platforms highlighted in this review, which primarily rely on enzymatic or chemical lysis to release SARS-CoV-2 nucleic acid [39,43,44]. One potential solution is to envision a separate POC device for mechanical lysis of non-sputum samples, provided the overall sample-to-result workflow remains user-friendly [45,46]. With efficient cell lysis, nucleic acid extraction could be skipped for tongue swabs, thereby simplifying the workflow [37,44].

Given the complexities associated with TB sample processing and the potential need for additional devices for lysis, adapting SARS-CoV-2 platforms for TB self-testing may be difficult, despite the authorization of several molecular SARS-CoV-2 tests for home-based testing [47,48].

High-yield sample lysis must pair with sensitive molecular detection methods. For instance, the frontrunner candidate, Lucira Check It, uses RT-LAMP, while the Visby COVID-19 Test employs RT-PCR. Currently, available isothermal amplification-based TB assays show high sensitivity but are limited in peripheral settings due to manual processes and outsourced DNA lysis and extraction [49,50]. Integrating these assays with sensitive POC platforms, as identified here, could streamline testing.

### 4.3. General Findings

Overall, we observed a lack of transparency in reporting instrument and test costs, with many exceeding WHO recommendations, when reported. Equitable access to WRDs remains elusive in LMICs despite large-scale investments and price negotiations [3,36,51]. Addressing global affordability and accessibility requires diversified manufacturing and minimized maintenance requirements [52]. However, most COVID-19 test manufacturers are based in high-income countries, hindering global access [52]. Translating these tests to TB will require a commitment from companies to global health and global access terms. SD Biosensor’s (Suwon, Republic of Korea) recent license agreement with the COVID-19 Technology Access Pool could serve as a model for TB diagnostics [53].

Many identified tests either lack independently reported clinical performance estimates or exhibit discrepancies between developer-reported and independently reported estimates, echoing recent findings on the overestimation of developer-reported sensitivity of SARS-CoV-2 antigen tests [54]. Moreover, standardized LoD reporting is necessary for meaningful comparisons between SARS-CoV-2 assays [55]. However, it is important to exercise caution when extrapolating LoD data from one pathogen to another.

### 4.4. Strengths

This scoping review has several strengths. Our systematic searches across various sources, including published studies, pre-prints, IVD databases, and manufacturers’ websites, ensure broad coverage of technologies from diverse developers, from start-ups to large IVD corporations. Data accuracy was ensured through rigorous screening by two independent reviewers and cross-verification with developer-reported information. Our TPP-aligned scorecard mitigates subjective reporting and can be readily applied to evaluate the potential adaptation of novel diagnostic tests emerging in future pandemics for TB. Additionally, focusing on commercially available, market-approved diagnostics may streamline the time-to-market for TB tests on these platforms.

### 4.5. Limitations

Several limitations should be noted. First, due to the extensive dataset and time constraint, charted data was not cross-verified by a second reviewer, and early-stage non-commercialized platforms were excluded from analysis, potentially overlooking promising POC technologies. Second, our search of IVD databases was confined to publicly accessible ones with English search functionality, which may introduce bias. Third, our scorecard needs further refinement, including weighing scores based on their relevance to end-users and incorporating additional TPP parameters, such as the current state of test deployment. Arguably, established platforms might score lower but could be more readily adopted by end-users than new platforms. Also, missing information was down-scored, which may have unfairly disadvantaged new platforms for which independently reported performance data was unavailable. Fourth, the scoring criteria were not specifically tailored to different technology classes. The scorecard, adapted to the 2014 TPPs for POC TB diagnostics, may be less applicable to low-complexity tests compared to (near)-POC tests. Fifth, data limitations, such as opaque cost reporting and a lack of clinical and analytical performance data, could bias device scoring and weaken the potential of identified tests to be effectively repurposed for TB. Moreover, the recent shutdown of some identified companies might result in the loss of promising platforms from the pipeline [56].

Lastly, the revised TPP was published only after our data analysis was completed. While we considered the draft version alongside the 2014 version during our analysis, we did not account for the specific requirements of different technology classes in our scoring. In addition, there are some minor differences in the revised TPPs, such as the reduction of the ‘maximum time-to-result’ from 120 to 60 min, and the ‘capital cost of equipment’ being capped at USD 2000. However, these changes do not affect the ranking of platforms within each technology class.

## 5. Conclusions

This scoping review highlights the potential for adapting SARS-CoV-2 POC diagnostic technologies for TB, identifying 66 commercially available antigen and molecular tests that may meet TPP criteria for peripheral settings. Platforms such as LumiraDx, Lucira Check-It, Visby, and Idylla, or those with similar features, should be prioritized for TB adaptation. The versatility of these technologies promises context-adapted tests that can integrate into local TB diagnostic algorithms. This study serves as a stepping stone toward leveraging COVID-19 diagnostics to bridge the TB diagnostic gap and urging stakeholders to collaborate on developing impactful TB diagnostic solutions.

## Data Availability

All relevant data have been included in the article or the Supplementary Materials. Additional raw data and analytical code can be accessed on OSF at https://osf.io/srwhj/?view_only=2e42118e87374da6b76e0344b0c17261 (accessed on 30 September 2024).

## Supplementary Materials

The following supporting information can be downloaded at: https://www.mdpi.com/article/10.3390/jcm13195894/s1, Section S1. R code for the medrxivr package; Table S1. PRISMA-ScR Checklist; Table S2. Data Charting Form; Table S3. Product Information Form; Table S4. Scorecard to evaluate the potential application of SARS-CoV-2 diagnostic devices in peripheral TB diagnosis (adapted from Lehe et al.); Table S5. Characteristics of Included Sources of Evidence and Charted Data Variables; Table S6. Near-POC Antigen Tests; Table S7. POC Molecular Tests; Table S8. Near-POC Molecular Tests; Table S9. Low-complexity Molecular Tests.
