# Supplementary material for: Innovative COVID-19 Point-of-Care Diagnostics Suitable for Tuberculosis Diagnosis: A Scoping Review
Source: J Clin Med. 2024 Oct 2;13(19):5894. doi: 10.3390/jcm13195894 (PMC11477317; doi:10.3390/jcm13195894)
Supplement: Supplementary file 1 [file jcm-13-05894-s001.zip › 4. Supplementary Materials_Scoping review.pdf]

Supplementary Materials:

Innovative COVID-19 Point-of-Care Diagnostics Suitable for Tuberculosis Diagnosis: A Scoping Review

Supplementary Methods

Supplementary Methods, Section 1. R code for the medrxivr package

```
#References: https://ropensci.org/blog/2020/10/20/searching-medrxivr-and-biorxiv-preprint-data/ ;
https://docs.ropensci.org/medrxivr/

# Set working directory
setwd("C:/Users/user/Documents/R/20220505 Rxiv Search")

# Install and load required packages
install_if_missing <- function(package_name) {
  if (!require(package_name, character.only = TRUE)) {
    install.packages(package_name, dependencies = TRUE)
    library(package_name, character.only = TRUE)}}

packages <- c("medrxivr", "dplyr", "ggplot2")
lapply(packages, install_if_missing)

# Load required libraries
library(medrxivr)
library(dplyr)
library(ggplot2)

# bioRxiv - Reproducible searching and exporting

# Retrieve preprint data within a specified date range from bioRxiv
preprint_data <- mx_api_content(server = "biorxiv", to_date = "2022-11-23")
```

Supplementary Materials

```
# Define search criteria
criteria <- list(
  condition = c("2019 nCoV", "2019nCoV", "2019 novel coronavirus", "COVID-19", "covid-19", "COVID19",
"covid 19", "new coronavirus", "novel coronavirus", "novel corona virus", "sars cov 2", "SARS-CoV-2",
"severe acute respiratory syndrome coronavirus 2"),
  tech = c("molecular", "isothermal", "PCR", "polymerase chain reaction", "LAMP", "CRISPR",
"immunoassay", "antigen"),
  setting = c("point of care", "POC", "near patient", "rapid test", "bedside test", "laboratory-independent",
"point-of-care", "POCT", "portable"),
  usecase = c("diagnos", "detect"))

# Run searches based on different criteria
results <- lapply(criteria, function(query) {
  mx_search(data = preprint_data, query = list(query))})

# Combine results
combined_results <- do.call(rbind, results)

# Export the combined results
mx_export(combined_results)
write.table(combined_results, "bioRxiv_data_20221123.txt", sep = ",", row.names = FALSE)
write.csv(combined_results, file = "bioRxiv_data_20221123.csv", sep = ",", row.names = FALSE)

# Download the PDF for each record
mx_download(combined_results)

# medrxiv - Reproducible searching and exporting

# Retrieve preprint data within a specified date range from medrxiv
preprint_data <- mx_api_content(server = "medrxiv", to_date = "2022-11-23")

# Define search criteria
criteria <- list(
  condition = c("2019 nCoV", "2019nCoV", "2019 novel coronavirus", "COVID-19", "covid-19", "COVID19",
"covid 19", "new coronavirus", "novel coronavirus", "novel corona virus", "sars cov 2", "SARS-CoV-2",
"severe acute respiratory syndrome coronavirus 2"),
  tech = c("molecular", "isothermal", "PCR", "polymerase chain reaction", "LAMP", "CRISPR",
"immunoassay", "antigen"),
  setting = c("point of care", "POC", "near patient", "rapid test", "bedside test", "laboratory-independent",
"point-of-care", "POCT", "portable"),
  usecase = c("diagnos", "detect"))
```

```
# Run searches based on different criteria
results <- lapply(criteria, function(query) {
  mx_search(data = preprint_data, query = list(query))})

# Combine results
combined_results <- do.call(rbind, results)

# Export the combined results
mx_export(combined_results)
write.table(combined_results, "medrxiv_data_20221123.txt", sep = ",", row.names = FALSE)
write.csv(combined_results, file = "medrxiv_data_20221123.csv", sep = ",", row.names = FALSE)

# Download the PDF for each record
mx_download(combined_results)
```

SUPPLEMENTARY TABLES

Table S1. PRISMA-ScR Checklist[1]

Preferred Reporting Items for Systematic reviews and Meta-Analyses extension for Scoping Reviews (PRISMA-ScR) Checklist

SECTION	ITEM	PRISMA-ScR CHECKLIST ITEM	REPORTED ON PAGE #
**TITLE**			
Title	1	Identify the report as a scoping review.	1
**ABSTRACT**			
Structured summary	2	Provide a structured summary that includes (as applicable): background, objectives, eligibility criteria, sources of evidence, charting methods, results, and conclusions that relate to the review questions and objectives.	1
**INTRODUCTION**			
Rationale	3	Describe the rationale for the review in the context of what is already known. Explain why the review questions/objectives lend themselves to a scoping review approach.	2

Objectives	4	Provide an explicit statement of the questions and objectives being addressed with reference to their key elements (e.g., population or participants, concepts, and context) or other relevant key elements used to conceptualize the review questions and/or objectives.	2
**METHODS**			
Protocol and registration	5	Indicate whether a review protocol exists; state if and where it can be accessed (e.g., a Web address); and if available, provide registration information, including the registration number.	2
Eligibility criteria	6	Specify characteristics of the sources of evidence used as eligibility criteria (e.g., years considered, language, and publication status), and provide a rationale.	2-3
Information sources*	7	Describe all information sources in the search (e.g., databases with dates of coverage and contact with authors to identify additional sources), as well as the date the most recent search was executed.	3
Search	8	Present the full electronic search strategy for at least 1 database, including any limits used, such that it could be repeated.	3
Selection of sources of evidence†	9	State the process for selecting sources of evidence (i.e., screening and eligibility) included in the scoping review.	3
Data charting process‡	10	Describe the methods of charting data from the included sources of evidence (e.g., calibrated forms or forms that have been tested by the team before their use, and whether data charting was done independently or in duplicate) and any processes for obtaining and confirming data from investigators.	3
Data items	11	List and define all variables for which data were sought and any assumptions and simplifications made.	3
Critical appraisal of individual sources of evidence§	12	If done, provide a rationale for conducting a critical appraisal of included sources of evidence; describe the methods used and how this information was used in any data synthesis (if appropriate).	Not applicable
Synthesis of results	13	Describe the methods of handling and summarizing the data that were charted.	4

**RESULTS**			
Selection of sources of evidence	14	Give numbers of sources of evidence screened, assessed for eligibility, and included in the review, with reasons for exclusions at each stage, ideally using a flow diagram.	4-5
Characteristics of sources of evidence	15	For each source of evidence, present characteristics for which data were charted and provide the citations.	6
Critical appraisal within sources of evidence	16	If done, present data on critical appraisal of included sources of evidence (see item 12).	Not applicable
Results of individual sources of evidence	17	For each included source of evidence, present the relevant data that were charted that relate to the review questions and objectives.	Supplementary materials (Tables S6 – S9)
Synthesis of results	18	Summarize and/or present the charting results as they relate to the review questions and objectives.	6-11
**DISCUSSION**			
Summary of evidence	19	Summarize the main results (including an overview of concepts, themes, and types of evidence available), link to the review questions and objectives, and consider the relevance to key groups.	12-14
Limitations	20	Discuss the limitations of the scoping review process.	14-15
Conclusions	21	Provide a general interpretation of the results with respect to the review questions and objectives, as well as potential implications and/or next steps.	14-15
**FUNDING**			
Funding	22	Describe sources of funding for the included sources of evidence, as well as sources of funding for the scoping review. Describe the role of the funders of the scoping review.	15

JBI = Joanna Briggs Institute; PRISMA-ScR = Preferred Reporting Items for Systematic reviews and Meta-Analyses extension for Scoping Reviews.

* Where *sources of evidence* (see second footnote) are compiled from, such as bibliographic databases, social media platforms, and Web sites.

† A more inclusive/heterogeneous term used to account for the different types of evidence or data sources (e.g., quantitative and/or qualitative research, expert opinion, and policy documents) that may be eligible in a scoping review as opposed to only studies. This is not to be confused with *information sources* (see first footnote).

Supplementary Materials

‡ The frameworks by Arksey and O'Malley (6) and Levac and colleagues (7) and the JBI guidance (4, 5) refer to the process of data extraction in a scoping review as data charting.
§ The process of systematically examining research evidence to assess its validity, results, and relevance before using it to inform a decision. This term is used for items 12 and 19 instead of "risk of bias" (which is more applicable to systematic reviews of interventions) to include and acknowledge the various sources of evidence that may be used in a scoping review (e.g., quantitative and/or qualitative research, expert opinion, and policy document).

Table S2. Data Charting Form

**Section #**	**Item #**	**Question type**	**Answer options**
**General study information**	#1 Study ID<br>#2 DOI<br>#3 Title<br>#4 First author<br>#5 Publication year<br>#6 Publication type<br>#7 Research type	#1 Short answer<br>#2 Short answer<br>#3 Short answer<br>#4 Short answer<br>#5 Drop-down<br>#6 Drop-down<br>#7 Drop-down	#1 NA<br>#2 NA<br>#3 NA<br>#4 NA<br>#5 2020, 2021, 2022<br>#6 Peer-reviewed, Pre-print<br>#7 Analytical research paper, Clinical research paper, Economic evaluation, Narrative review, Qualitative research paper, Systematic review, Unclear, Other
**Clinical performance**	#1 Study country<br>#2 Study design<br>#3 Study population<br>#4 Sample size<br>#5 Sensitivity (95% CI)<br>#7 Specificity (95% CI)<br>#8 Conflict of interest<br>#9 Financial support<br>#10 Developer among authors	#1 Drop-down<br>#2 Drow-down<br>#3 Drow-down<br>#4 Short answer<br>#5 Short answer<br>#6 Short answer<br>#7 Short answer<br>#8 Drop-down<br>#9 Drop-down<br>#10 Drop-down	#1 Afghanistan (...) Zimbabwe, Multi-country, Not reported, Not relevant<br>#2 Case-control, Case report/series, Cross-sectional, RCT, Systematic review, Unclear, Other<br>#3 Adults, Children, Mixed, Not reported<br>#4 NA<br>#5 NA<br>#6 NA<br>#7 NA<br>#8 Yes, No, Unclear<br>#9 Yes, No, Unclear<br>#10 Yes, No, Unclar
**Test characteristics**	#1 Developer<br>#2 Country<br>#3 Product name<br>#4 Alternative product name<br>#5 Product type<br>#6 Product category<br>#7 Product description<br>#8 Limit of detection (copies/mL)<br>#9 Sample type<br>#10 Sample preparation<br>#11 Footprint (mm)	#1 Short answer<br>#2 Drop-down<br>#3 Short answer<br>#4 Short answer<br>#5 Drop-down<br>#6 Short answer<br>#7 Short answer<br>#8 Short answer<br>#9 Short answer<br>#10 Drop-down<br>#11 Short-answer	#1 NA<br>#2 Afghanistan (...) Zimbabwe<br>#3 NA<br>#4 NA<br>#5 Antigen test, Molecular test<br>#6 NA<br>#7 NA<br>#8 NA<br>#9 NA<br>#10 No manual steps, 1-2 steps, >2 steps, Unclear<br>#11 NA<br>#12 Yes, No, Unclear

#12 Multi-use platform	#12 Drop-down	#13 NA
#13 Throughout capacity	#13 Short answer	#14 NA
#14 Time-to-result (min)	#14 Short answer	#15 NA
#15 Hands-on time (min)	#15 Short answer	#16 Yes, No, Unclear
#16 Connectivity	#16 Drop-down	#17 25, 30, 40, 50, Unclear, Other
#17 Max operating	#17 Drop-down	#18 70, 90, Unclear, Other
temperature (degree celsius)	#18 Drop-down	#19 12, 24, Unclear, Other
#18 Max operating humidity	#19 Drop-down	#20 Standard electricity, Solar-powered, Battery-
(%)	#20 Drop-down	powered, None, Unclear, Other
#19 Shelf-life	#21 Drop-down	#21 <$1, $1-$2, $2-$4, $4-$10, >$10, Unclear, Other
#20 Power requirement	#22 Drop-down	#22 <$20, $20-$100, $100-$500, $500-$1000, $1000-$5000,
#21 Test price ($)		>$5000, Unclear, not applicable (instrument-free), Other
#22 Instrument price ($)		

***Abbreviations**: NA = Not applicable; CI = Confidence Interval*

Table S3. Product Information Form

**Item #**	**Question type**	**Answer options**
#1 Reference source	#1 Checkboxes	#1 Developer website, FDA, EUDAMED, NMPA,
#2 Developer	#2 Short answer	MFDS, MDALL, CDSCO, FIND, Johns Jopkins, RADx
#3 Developer website	#3 Short answer	#2 NA
#4 Business type	#4 Dropdown	#3 NA
#5 Country	#5 Drop-down	#4 Academia, Start-up, Not-for-profit, Micro company
#6 Product name	#6 Short answer	(<10), Small-sized company (10-49), Medium-sized
#7 Product description	#7 Short answer	company (50-250), Large-sized company (>250)
#8 Product type	#8 Drop-down	#5 Afghanistan (...) Zimbabwe
#9 Product category	#9 Checkboxes	#6 NA
#10 Sample type	#10 Checkboxes	#7 NA
#11 Sample preparation	#11 Drop-down	#8 Antigen test, Molecular test
#12 Sensitivity (95% CI)	#12 Short answer	#9 Automated immunoassay, CRISPR, High-throughput
#13 Specificity (95% CI)	#13 Short answer	molecular test, Isothermal amplification, Rapid
#14 Limit of detection	#14 Short answer	biosensor, Rapid molecular platform, Reader-based
(copies/mL)	#15 Drop-down	lateral flow assay, Vertical flow assay, Other
#15 Test price ($)	#16 Drop-down	#10 Breath, Bronchoalveolar lavage, Feces, Mid-
#16 Instrument price ($)	#17 Short answer	turbinate swab, Nasal swab, Nasopharyngeal sample,
#17 Footprint (mm)	#18 Drop-down	Oropharyngeal sample, Purified RNA, Saliva, Sputum,
#18 Multi-use platform	#19 Short answer	Whole blood, Other
#19 Throughput capacity	#20 Short answer	#11 No manual steps, 1-2 steps, >2 steps, Unclear
#20 Time-to-result (min)	#21 Short answer	#12 NA
#21 Hands-on time (min)	#22 Drop-down	#13 NA
#22 Connectivity	#23 Drop-down	#14 NA
#23 Max operating	#24 Drop-down	#15 <$1, $1-$2, $2-$4, $4-$10, >$10, Unclear, Other
temperature (degree celsius)	#25 Drop-down	#16 <$20, $20-$100, $100-$500, $500-$1000, $1000-$5000,
#24 Max operating humidity	#26 Drop-down	>$5000, Unclear, Not applicable (instrument-free), Other
(%)	#27 Drop-down	#17 NA
#25 Shelf-life	#28 Drop-down	#18 Yes, No, Unclear
#26 Power requirements	#29 Checkboxes	#19 NA
#27 Technology readiness	#30 Drop-down	#20 NA

level (TRL)<br>#28 Regulatory status<br>#29 Potential use case<br>#30 Potential end user	#21 NA<br>#22 Yes, No, Unclear<br>#23 5, 30, 40, 50, Unclear, Other<br>#24 70, 90, Unclear, Other<br>#25 12, 24, Unclear, Other<br>#26 Standard electricity, Solar-powered, Battery-powered, None, Unclear, Other<br>#27 TRL1 (...) TRL9, Unclear<br>#28 CE-marked, Korea MFDS, US FDA EUA, US FDA 510k, WHO EUL, WHO-endorsed, Research use only, Under development, Unclear<br>#29 Detection, Triage, Drug susceptibility testing<br>#30 Trained laboratory technician, Healthcare workers trained to the level of auxiliary nurses, Healthcare workers with a minimum of training, Community or healthcare workers with a minimum of training, Self-testing, Other

Abbreviations: *NA = Not applicable; CRISPR = Clustered Regularly Interspaced Short Palindromic Repeats*

Table S4. Scorecard to evaluate the potential application of SARS-CoV-2 diagnostic devices in peripheral TB diagnosis (adapted from *Lehe et al*)[2]

Scoring category	Scoring criteria	Scoring variables	Definitions	Specifications	Scoring thresholds
**1 PoC features of equipment**	Technical specifications	Instrument size	*Instrument size (cm).*	None	Disposable = 5<br>Handheld = 3<br>Tabletop = 1
		Instrument weight	*Instrument weight (kg)*	None	Could be transported by hand (<5 kg) = 5<br>Could be transported by vehicle (<15 kg) = 3<br>Could not be transported (>15 kg) =1
		Power requirements	*Power source required to run the diagnostic device.*	None	Instrument-free = 5<br>Instrument with optional battery-powered operation = 4<br>Instrument with optional solar-powered operation = 3<br>Instrument running on mains electricity plus an uninterrupted power supply unit = 2<br>Instrument running on mains electricity only = 1
		Instrument-free	*Requirement of an instrument to run the diagnostic assay.*	None	Not required=5<br>Required=1
		Connectivity	*The diagnostic device can be connected to external devices, databases and/or cloud services via wifi or bluetooth connectivity. No manual data transfer via USB stick or other means is required.*	None	True = 5,<br>False = 1
	Data analysis	Integrated data analysis	*Data analysis and result display are integrated into the diagnostic device and do not require external devices.*	None	True = 5,<br>False = 1

		Integrated electronics and software	*The electronics and software required to process and display the test data are integrated into the diagnostic device.*		True = 5,<br>False = 1
	Testing capacity	Time-to-result	*Time passed between sample collection and result display (minutes).*	*When a range was reported, the upper limit was used as the reference value for scoring purposes.*	<15min = 5<br><30 min = 4<br><1 hour = 3<br><2 hours = 2<br>>= 2 hours = 1
		Hands-on-time	*Time passed between sample collection and automated sample processing by the diagnostic device (minutes).*	*When a range was reported, the upper limit was used as the reference value for scoring purposes.*	<1 min = 5<br><5 min = 4<br><10 min = 3<br>>10 min = 1
		Throughput capacity	*Number of samples that can be processed by the diagnostic device in a single run.*	None	≥ 2 tests per run = 5<br>1 test per run = 1
**2 PoC features of test consumables**	Operating conditions	Operating Temperature	*The maximum allowable ambient temperature (°C) at which the diagnostic device can be operated.*	None	Up to 50°C = 5<br>Up to 40°C = 4<br>Up to 30°C = 3<br>Up to 25°C = 2<br><25°C = 1
		Operating Humidity	*The maximum allowable ambient humidity (%) at which the diagnostic device can be operated.*	None	Up to 90% humidity = 5<br>Up to 70% humidity = 3<br><70% humidity = 1
	Storage conditions	Shelf life	*The duration (months) for which the diagnostic assay can be stored without becoming unfit for diagnostic testing, i.e. without compromising on accuracy.*	None	>12 Months = 5<br>6–12 Months = 3<br><6 Months = 1

**3 Ease of use**	End user requirements	Potential end user	*Level of professional training required of the device's potential end user.*	*1. Community or lay health worker without technical skills: diagnostic devices for which no special technical skills are required, including those suitable for self-testing*<br><br>*2. Healthcare workers with basic technical skills: diagnostic devices that require basic technical skills. Devices in this category require pre-processing of the sample that can be performed without laboratory equipment.*<br><br>*3. Healthcare workers with more advanced technical skills: diagnostic devices that require more advanced technical skills. Devices falling into this category require extensive sample pre-processing including laboratory equipment, such as exact pipetting and precise volume transfer.*	Community/lay health worker without technical skills = 5<br>Healthcare worker with basic technical skills = 3<br>Healthcare worker with more advanced technical skills = 1
		Number of Manual Sample Processing Steps	*The number of sample processing steps that must be performed manually between sample collection and automated sample processing by the diagnostic device. This excludes the sample collection itself, but includes steps such as pipetting, addition of buffers, or vortexing.*	None	No manual steps = 5<br>1-2 steps = 3<br>> 2 steps = 1
**4 Performance**	Analytical and clinical performance (COVID-19)	Limit of detection (LoD)	*The smallest concentration of analyte that can be detected by the diagnostic assay. For scoring purposes, the lowest reported LoD (copies/mL) was used.*	*Only study-reported estimates were considered even if a developer-reported value could be obtained.*	≤ 100 copies/mL = 5<br>≤ 500 copies/mL = 3<br>> 500 copies/mL = 1

		Clinical sensitivity	*The probability of a positive test results in truly positive individuals.*	*If available, estimates from systematic reviews were used. If only estimates from cross-sectional studies or case-control studies were available, estimates from cross-sectional studies were preferred. If multiple estimates were available, the estimate from the study with the largest sample size was selected. Only study-reported estimates were considered even if a developer-reported value could be obtained.*	> 98 % = 5<br>> 95 % = 3<br>< 95 % = 1
		Clinical specificity	*The probability of a negative test result in truly negative individuals.*	*If available, estimates from systematic reviews were used. If only estimates from cross-sectional studies or case-control studies were available, estimates from cross-sectional studies were preferred. If multiple estimates were available, the estimate from the study with the largest sample size was selected. Only study-reported estimates were considered even if a developer-reported value could be obtained.*	> 98 % = 5<br>> 95 % = 3<br>< 95 % = 1
**5 Cost**	Upfront and user costs	Capital cost of equipment	*Fixed, one-time upfront costs that are incurred when purchasing the diagnostic device ($).*	*Instrument-free diagnostics received a score of 5 since this parameter does not apply to them.*	<$1,000 = 5<br>$1,000–5,000 = 3<br>>$5,000 = 1
		Consumable cost	*Recurring costs to run a single diagnostic test ($)*	None	<$2 =5<br>$2–10 per test = 3<br>>$10 per test =1

**6 Platform versatility**	Multi-use ability	Platform support for multi-use tests	*The ability to analyze multiple biomarkers from one sample on a single diagnostic device. Such multi-disease panels can for example include respiratory assays that detect a range of different respiratory pathogens from a single sample.*	None	True = 5<br>False = 1
**7 Parameters**	Test parameters	Number of test parameters available for scoring	*Number of test parameters available to use for scoring purposes (x out of 21 parameters) for each given diagnostic device. For example, if no clinical sensitivity value was available for a given diagnostic device, only 20/21 variables could be scored.*	None	>18 parameters available = 5<br>12-18 parameters available = 3<br><12 parameters available = 1

Abbreviations*: PoC = Point-of-Care; NA = Not applicable*

Table S5. Characteristics of Included Sources of Evidence and Charted Data Variables

Source of evidence and article type		Charted variables	Comments
Primary sources of evidence	Analytical research paper	• Focus on the development of new diagnostic tools, with predominantly proof-of-concept studies and early development of platforms for diagnostic assays;<br>• Early-stage, pilot performance studies in a small number of participants;<br>• Price estimates were rarely provided	• Seldom contained data on commercialized diagnostic devices, focus on products in the early stages of development
	Clinical research paper	• (Pooled) test performance of commercialized diagnostic tests, comparing performance of the diagnostic test under investigation against the gold-standard for SARS-CoV-2 diagnosis, RT-PCR.	• Source of study-reported performance data
	Systematic review	• Range of or pooled test performance;<br>• Little further information on developer or diagnostic test	• Source of range of pooled performance data
	Narrative review	• Description of a range of diagnostic tests falling within the scope of the review topic, including technical details of the diagnostic test and operational characteristics;<br>• Clinical performance (mostly developer-reported);<br>• Pricing was rarely mentioned	• Performance data was often extracted from the developer's website
Secondary sources of evidence	Developer website	• Supplementary data on diagnostic assays and instruments, in particular information on technical details, workflow, costs, and end user requirements;<br>• Developer-reported performance data	• Discrepancies between developer-reported and study-reported performance data
	Regulatory agencies	• Identification of instructions for use (IFU) documentation, which provided complementary information on assays and diagnostic device characteristics;<br>• Regulatory status in the respective countries	• Main source for standardized, complete information on diagnostics approved tests
	SARS-CoV-2 test databases: FIND COVID-19 Test Directory[a], Johns Hopkins COVID-	• Overview of regulatory status globally, performance data, validated sample types and target analyte	• Main source used to obtain information on the regulatory status of diagnostic devices globally, and to link products to country-specific databases

19 Testing Toolkit[b], and NIH RADx[c]

Abbreviations: *RT-PCR = Reverse-Transcriptase Polymerase Chain reaction; IFU = Instructions For Use; NIH RADx = National Institutes of Health Rapid Acceleration of Diagnostics.*
Legend: [a]*FIND COVID-19 Test Directory: https://www.finddx.org/tools-and-resources/dxconnect/test-directories/covid-19-test-directory/*
[b]*Johns Hopkins COVID-19 Testing Toolkit: https://covid19testingtoolkit.centerforhealthsecurity.org/testing-trackers/antigen-and-molecular-tests-for-covid-19*
[c]*NIH RADx: https://www.nibib.nih.gov/covid-19/radx-tech-program/authorized-tests*

Table S6. Near-POC Antigen Tests.

[Please see separately attached excel file]

Table S7. POC Molecular Tests.

[Please see separately attached excel file]

Table S8. Near-POC Molecular Tests.

[Please see separately attached excel file]

Table S9. Low-complexity Molecular Tests.

[Please see separately attached excel file]

References

1 Tricco AC, Lillie E, Zarin W, O'Brien KK, Colquhoun H, Levac D, et al. PRISMA Extension for Scoping Reviews (PRISMAScR): Checklist and Explanation. Ann Intern Med. 2018;169:467–473. doi: 10.7326/M18-0850

2 Lehe, J.D., et al., *Evaluating operational specifications of point-of-care diagnostic tests: a standardized scorecard.* PLoS One, 2012. 7(10): p. E47459
